# Regulation of intestinal stem cell activity by a mitotic cell cycle regulator Polo in *Drosophila*

**DOI:** 10.1093/g3journal/jkad084

**Published:** 2023-06-01

**Authors:** Ying Zhang, Rongbing Chen, Liyuan Gong, Wuren Huang, Ping Li, Zongzhao Zhai, Erjun Ling

**Affiliations:** Research Center for Translational Medicine at Shanghai East Hospital, School of Life Sciences and Technology, Tongji University, Shanghai 200092, China; CAS Key Laboratory of Insect Developmental and Evolutionary Biology, CAS Center for Excellence in Molecular Plant Sciences, The Chinese Academy of Science, Shanghai 200032, China; University of Chinese Academy of Sciences, Beijing 101408, China; CAS Key Laboratory of Insect Developmental and Evolutionary Biology, CAS Center for Excellence in Molecular Plant Sciences, The Chinese Academy of Science, Shanghai 200032, China; University of Chinese Academy of Sciences, Beijing 101408, China; CAS Key Laboratory of Insect Developmental and Evolutionary Biology, CAS Center for Excellence in Molecular Plant Sciences, The Chinese Academy of Science, Shanghai 200032, China; University of Chinese Academy of Sciences, Beijing 101408, China; CAS Key Laboratory of Insect Developmental and Evolutionary Biology, CAS Center for Excellence in Molecular Plant Sciences, The Chinese Academy of Science, Shanghai 200032, China; Research Center for Translational Medicine at Shanghai East Hospital, School of Life Sciences and Technology, Tongji University, Shanghai 200092, China; Hunan Provincial Key Laboratory of Animal Intestinal Function and Regulation, College of Life Sciences, Hunan Normal University, Changsha 410081, China; CAS Key Laboratory of Insect Developmental and Evolutionary Biology, CAS Center for Excellence in Molecular Plant Sciences, The Chinese Academy of Science, Shanghai 200032, China

**Keywords:** intestinal stem cell, Polo, mitosis, aneuploidy, differentiation, apoptosis

## Abstract

Maintaining a definite and stable pool of dividing stem cells plays an important role in organ development. This process requires an appropriate progression of mitosis for proper spindle orientation and polarity to ensure the ability of stem cells to proliferate and differentiate correctly. Polo-like kinases (Plks)/Polo are the highly conserved serine/threonine kinases involved in the initiation of mitosis as well as in the progression of the cell cycle. Although numerous studies have investigated the mitotic defects upon loss of Plks/Polo in cells, little is known about the in vivo consequences of stem cells with abnormal Polo activity in the context of tissue and organism development. The current study aimed to investigate this question using the *Drosophila* intestine, an organ dynamically maintained by the intestinal stem cells (ISCs). The results indicated that the *polo* depletion caused a reduction in the gut size due to a gradual decrease in the number of functional ISCs. Interestingly, the *polo-*deficient ISCs showed an extended G2/M phase and aneuploidy and were subsequently eliminated by premature differentiation into enterocytes (ECs). In contrast, the constitutively active Polo (*polo^T182D^*) suppressed ISC proliferation, induced abnormal accumulation of β-tubulin in cells, and drove ISC loss via apoptosis. Therefore, Polo activity should be properly maintained for optimal stem cell function. Further analysis suggested that *polo* was a direct target gene of Sox21a, a Sox transcription factor that critically regulates stem cell activity. Together, this study provided a novel perspective on the correlation between the progression of mitosis and the ISC function in *Drosophila*.

## Introduction

Polo kinases, belonging to a highly conserved serine/threonine (Ser/Thr) kinase family, were first found in *Drosophila melanogaster* (Sunkel and Glover 1988), which consist of a canonical Ser/Thr kinase domain and a unique Polo box domain that enable the kinases to bind to their target proteins (Lowery et al. 2005; Alvarez-Rodrigo et al. 2021). Five Polo-like kinases (Plks) have been identified in mammals, including Plk1, Plk2/SNK, Plk3/CNK/FNK, Plk4/SAK, and Plk5 (de Cárcer et al. 2011; Goroshchuk et al. 2019). *Drosophila* and budding yeast have only 1 Plk, known as Polo and Cdc5, respectively (Archambault and Glover 2009). Polo kinases are activated by phosphorylation (Tavares et al. 1996; Peng et al. 2021). Plk1 and Polo are activated by aurora A and aurora B, respectively (Macůrek et al. 2008; Carmena et al. 2012). Thr182 is a major phosphorylation site in *Drosophila* Polo (Carmena et al. 2012), and T210 is the corresponding site in Plk1 (Jang et al. 2002; Kachaner et al. 2014). Polo kinases play multiple roles in the cell cycle, including mitotic entry, centrosome organization, spindle formation, anaphase-promoting complex regulation, mitotic exit, and cytokinesis (Barr et al. 2004; Archambault and Glover 2009; Gallaud et al. 2022; Wong et al. 2022).

The studies on Polo have mainly focused on its role in regulating the cell division of embryos, brain cells, and neuroblasts. Mutations in the *polo* gene can cause a series of problems in mitosis, such as prolonging mitotic duration, affecting spindle orientation, and inducing chromosome misalignment (Peng et al. 2019; Gallaud et al. 2022). In the brain cells with *polo^9/10^*, in which Polo kinases were barely detectable (Donaldson et al. 2001), the number of neuroblasts increased (Wang et al. 2007). The spindle assembly checkpoint (SAC) and Polo can protect neural stem cells (NSCs) from undergoing excessive proliferation and aneuploidy (Gallaud et al. 2022). Numerous nonmitotic functions of Plk1/Polo, including DNA damage response, autophagy, and neurological disorders, such as Alzheimer’s disease and Huntington's disease, have also been reported (Peng et al. 2015; Li et al. 2017; Mo et al. 2019). Polo was also expressed in the midgut of *Drosophila* and enriched in intestinal stem cell (ISCs) as compared with the other cell types (Dutta et al. 2015; Doupé et al. 2018). However, little is known about its in vivo function in the development of *Drosophila* midgut. In addition, *polo* has been implicated as both an oncogene and a tumor suppressor gene depending on the context (de Cárcer 2019; Gallaud et al. 2022); therefore, its role in tumors remains controversial.

In order to genetically stabilize daughter cells, a euploid genome should be maintained in the stem cells (Matzke et al. 1999; Tolmacheva et al. 2020). The gain or loss of the whole chromosome caused by errors during mitosis leads to aneuploidy, resulting in developmental disorders and finally death (Siegel and Amon 2012; Ben-David and Amon 2020). Therefore, apoptosis occurs in aneuploid cells and organisms (Thompson and Compton 2010; Singla et al. 2020). As compared with the other proliferative cells (Morais da Silva et al. 2013), studies indicated that adult stem cells might tolerate aneuploidy instead of activating apoptosis (Mantel et al. 2007; Harper et al. 2010). Subsequently, aneuploidy could induce the differentiation of ISCs (Gogendeau et al. 2015) or promote their proliferation and gut dysplasia (Resende et al. 2018; Brás et al. 2021) in *Drosophila*.

**Fig. 1.**
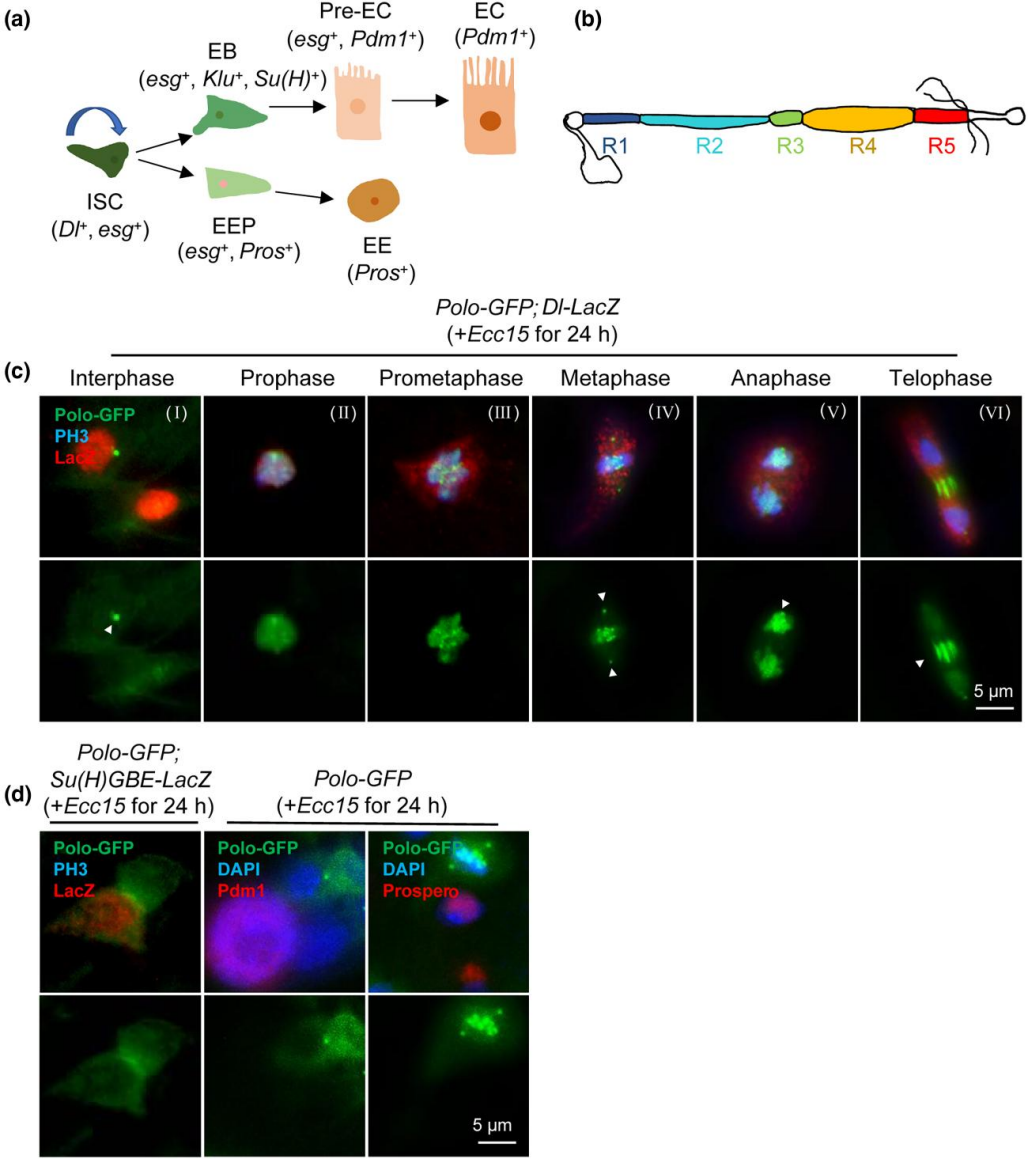
Polo was specifically expressed in progenitor cells and showed a dynamic distribution during mitosis. a) A schematic for the ISC lineage in adult *Drosophila* midgut. ISCs (marked by *Dl*) could self-renew and generate 2 types of committed progenitors EBs (marked by *Su(H)* and *Klu*) and EEPs (marked by low levels of both *Dl* and *Pros*). The EBs and EEPs respectively developed into ECs (marked by *Pdm1*) and EEs (marked by *Pros*). b) Surface view of 5 regions (R1, R2, R3, R4, and R5) of *Drosophila* midgut. Unless otherwise noted, we focused our study on the specific R4 region of the posterior midgut. c) Dynamic localization of Polo-GFP during cell division at different phases of mitosis. Adult flies with the indicated genotypes were orally infected with *Ecc15* to induce cell division in their midguts. Immunostaining labeled the intestinal cells undergoing cell division using an antibody against PH3 and ISC marker *Dl-LacZ*, respectively. The arrows indicate the puncta in interphase cells and spindle poles, central spindle in mitotic cells. Scale bar, 5 *µ*m. d) Polo was weakly expressed in EBs. No Polo signal was detected in ECs and EEs. EB was identified using the line of *Su(H)GBE-LacZ*. EC and EE were identified using the antibody against Pdm1 and Prospero, respectively. Scale bar, 5 *µ*m.

The intestine of *Drosophila* is composed of ISCs, enteroblasts (EBs), enterocytes (ECs), and enteroendocrine cells (EEs) (Ohlstein and Spradling 2006). It is an excellent in vivo study model for adult stem cell behavior, in which, all the cell types can be identified by their specific markers such as *Dl* (ISC), *Su(H)* and *klu* (EB), *Pdm1* (EC), and *Pros* (EE) (Ohlstein and Spradling 2007; Korzelius et al. 2019; Reiff et al. 2019; Zipper et al. 2020) (Fig. 1a). The expression levels of genes in specific cell types can be manipulated using a series of genetic tools (Jiang and Edgar 2012). The *Drosophila* midgut was divided into 5 main regions that were named R1–R5 (Buchon et al. 2013; Bohere et al. 2022). The current study focused on the specific R4 region of the posterior midgut (Fig. 1b).

The current study utilized the *Drosophila* midgut to explore the proliferation and differentiation of ISCs caused by mitotic defects using loss of function and gain of function of Polo. The results showed that the *polo-*deficient ISCs were correlated with an extended G2/M phase and aneuploidy and were subsequently lost by premature differentiation into ECs. In contrast, the constitutively active Polo (*polo^T182D^*) suppressed ISC proliferation, induced abnormal accumulation of β-tubulin in cells, and drove ISC loss via apoptosis. Thus, the Polo activity should be properly maintained for optimal stem cell function in the *Drosophila* midgut.

## Materials and methods

### Fly lines and husbandry

The following fly lines were used:

*esg^ts^*: *esg-Gal4*, *UAS-GFP*, *tub-Gal80^ts^* (Ⅱ) (Micchelli and Perrimon 2006);

*Su(H)GBE^ts^*: *Su(H)GBE-Gal4*, *UAS-GFP*, *tub-Gal80^ts^**/**Cyo* (Zeng et al. 2010);

*ISC^ts^*: *esg-Gal4*, *UAS-eYFP*; *Su(H)-Gal80*, *tub-Ga80^ts^**/**TM3 sb* (Wang et al. 2014);

*esg^ReDDM^*: *esg-Gal4*, *UAS-CD8::GFP**/**cyo; tub-Gal80^ts^*, *UAS-H2B::RFP**/**TM2* (Antonello et al. 2015);

*y*: *hs-Flp*, *tub-Gal4*, *UAS-nls GFP;;tub-Gal80*, *FRT80B**/**TM6B*;

*Dl^ts^*: *Dl-Gal4*, *tub-Gal80^ts^**/**TM6B*;

*polo^1^*; *UAS-polo-IR*; *UAS-polo^T182D^*; *UAS-polo.ORF.3×HA*; *Polo-GFP*; *Sox21a^6^*; *UAS-Sox21a*; *UAS-fzy-IR*; *UASt-NLS-GFP-E2F1*, *UASt-NLS-RFP-CycB* (Zielke et al. 2014); *UAS-p35*; *hid-LacZ*; *Dl-LacZ*; *Su(H)GBE-LacZ*; *FRT80B*.

A full list of genotypes used in each experiment is shown in Supplementary Table 1.

Female flies were used in all experiments. All flies were fed on a standard medium (maize flour, dead yeast, and agar) at room temperature, unless otherwise mentioned. In most cases, the drive lines (*esg^ts^*, *Dl^ts^*, *Su(H)GBE^ts^*, *ISC^ts^*, and *esg^ReDDM^*) were crossed with *w^1118^* or the UAS-linked transgene for *polo* knockdown or *polo* overexpression.

### Immunohistochemistry

Adult midguts were dissected in 0.85% NaCl, fixed at 4°C overnight in fixative solution (P0098, Beyotime), washed in PBST [PBS containing 3% bovine serum albumin (BSA) and 0.1% Triton X-100] 3 times, and then incubated in primary antibodies (at 4°C overnight) and secondary antibody (at 25°C for 4 h or 4°C overnight) prepared in PBST. All antibodies were listed in Supplementary Table 1. Nuclei were counterstained with 4′,6-diamidino-2′-phenylindole dihydrochloride (DAPI) (D9542, Sigma). All images were taken on the SP8 STED Confocal Microscope or Olympus BX51 fluorescent microscope. Images were processed and quantified using ImageJ software.

### Oral infection with *Ecc15* and colchicine feeding

*Erwinia carotovora carotovora 15* (*Ecc15*) was grown in LB medium at 29°C with shaking overnight. On next day, *Ecc15* cells were harvested after centrifuging at 5,000 rmp at 4°C for 5 min. The pellet was then washed using 0.85% NaCl and 5% sucrose respectively at the same condition. Finally, *Ecc15* cells were suspended with 5% sucrose to OD_600_ = 200 for oral infection. The control flies were fed with 5% sucrose. Colchicine was prepared as reported previously (Guo et al. 2013). Approximately 200 *µ*g/ml colchicine (HY-16569, MCE) was suspended with 5% sucrose for feeding flies of different genotypes for 24 h. The control flies were fed with 5% sucrose. All flies were dry-starved in the empty tubes for 2 h before feeding.

### Conditional expression of UAS-linked transgene

The TARGET system was performed in combination with the gal4-driver (*esg-gal4*, *Su(H)GBE-gal4*, and *Dl-gal4*) to conditionally express UAS-liked transgenes (McGuire et al. 2003). Flies were fed at 18°C to limit gal4 activity for 3–4 d and then shifted to 29°C for different time to inactivate the temperature-sensitive Gal80's activity to induce transgene expression. Combined with TARGET system, this tool allows the UAS-linked transgene to express in cells with GFP expressed (Lee and Luo 1999).

### MARCM clone induction

Mosaic analysis with a repressible cell marker (MARCM) technique was used for clonal analysis (Lee and Luo 2001). Transgenes were only expressed in the clones indicated by the presence of GFP. 3-5-day-old flies with the appropriate genotypes were maintained at 25°C for 7 d after heat-shocked at 37°C for 30 min in a water bath. Experiments were performed by combining with the *FRT80B*, *polo^1^*, and *UAS-polo^T182D^*.

### TUNEL staining

The dissected midguts were fixed as above and permeabilized using 2 mg/ml Proteinase K for 7 min, followed by washing and incubation in the dark for 1 h at 37°C in 100-*µ*l TUNEL reaction mixture (In Situ Cell Death Detection kit, TMR Red, Roche) as described in the manufacturer's protocol.

### Fluorescence in situ hybridization

The described fluorescence in situ hybridization (FISH) protocol was followed with some modification (Dernburg 2011; Gogendeau et al. 2015; Resende et al. 2018). Briefly, oligonucleotide probe for dodecaheterochromatic repeats (chromosome III) was synthesized with a 5′-Cy5 by the integrated DNA technologies. The following sequence on the chromosome III was used to prepare the probe: 5′-Cy5 CCCGTACTGGTCCCGTACTGGTCCCG-3′. The intestines from female flies were dissected and fixed in 4% paraformaldehyde at 4°C overnight, washed 3 times in PBST, followed by another 2 washing in 2×SSCT and 2×SSCT/50% formamide respectively. For prehybridization, intestines were transferred to a PCR tube containing 92°C prewarmed 2×SSCT/50% formamide and denatured for 3 min at 92°C. Midguts were then hybridized with the above denatured DNA probe (40–80 ng) in hybridization buffer at 92°C for 3 min, in which there were 20% dextran sulfate (D8906, Sigma), 2×SSCT/50% deionized formamide (30091218, CAS), and 0.5 mg/ml salmon sperm DNA (H1060, Solarbio). After that, the tubes were incubated at 37°C overnight. Samples were then washed with 2×SSCT prewarmed at 60°C for 10 min and finally in the buffer of 2×SSCT and PBS at room temperature for 5 min. To identify GFP-labeled ISCs/EBs, the above midguts were incubated with an anti-GFP antibody at 4°C overnight, followed by incubation in the secondary antibody at 4°C overnight. Samples were then washed using PBST 3 times and incubated in PBS containing DAPI. GFP-labeled cells were scored as aneuploid when more than 2 dots of the respective FISH probes were detected.

### Luciferase assays

S2 cells were cultured as previously described (Guo et al. 2020). Luciferase reporters were generated through cloning the genomic DNA sequence of *polo* promoter fragments (as described in Fig. 6d) into pGL3-basic vector containing the hsp70 basal promoter. S2 cells were transfected with the above luciferase reporters and internal control Copia–Renilla vector, along with pAc-GFP as control or pAc-Sox21a plasmid to overexpress Sox21a for Fig. 6f. Cells were incubated for 48 h after transfection. Luciferase activities were assessed by Dual-Luciferase Reporter Assay system (E1910, Promega). The primers were listed in Supplementary Table 2.

### Expression of Sox21a^HMG-BD^

The sequence of high mobility group box domain (HMG-BD) of Sox21a (named as Sox21a^HMG-BD^, from 108–208 aa) was cloned and inserted into the expression vector pET-28b (Novagen) with a His-tag at the C-terminals. The recombinant Sox21a^HMG-BD^ was expressed and purified as described (Li et al. 2012b). The protein concentration was determined using BCA Protein Assay Kit (Epizyme). The purified protein in 5% glycerol was aliquoted and stored at −80°C. Sox21a-bound domain sequence was listed in Supplementary Table 3. The primers were listed in Supplementary Table 2.

### Electrophoretic mobility shift assay

The described electrophoretic mobility shift assay (EMSA) protocol was followed with some modification (Ma et al. 2020). Briefly, Sox21a^HMG-BD^ was purified as described above. The Cy5-labeled probe (F2-4) was amplified using 2 rounds of PCR. The Cy5-labeled F2-4-3 probe containing the wild-type sequence and the probe containing mutated sequence were synthesized by Biosune. The Cy5-labeled probe and protein were coincubated in EMSA–binding buffer (25 mM HEPES, pH 7.5, 40 mM KCl, 3 mM DTT, 10% glycerol, 0.1 mM EDTA, 0.5 mg/ml BSA, 0.5 mg/ml poly-glutamate) at 25°C for 30 min in dark. After incubation, the reaction mixture was electrophoresed on a 6% native polyacrylamide gel, and then labeled DNA was detected using Typhoon FLA scanners (Amersham Typhoon 5, Cytiva). The sequences of probes were listed in Supplementary Table 3. The primers were listed in Supplementary Table 2.

### qRT-PCR analysis of gene expression

Total RNA was extracted from 30 midguts per example using TRIzol reagent (Invitrogen). cDNA was synthesized using the Prime Script RT reagent kit (Toyobo). Total RNA (1 *µ*g) was applied for reverse transcription with oligo dT, and the first strand cDNA was diluted with water for further use in real-time PCR as described (Tang et al. 2021). The expression in control sample was normalized to 1. Primers for detecting gene expression were listed in Supplementary Table 2.

### Lifespan analysis

Genetic crosses were set up to overexpression active *polo^T182D^* driven by *esg^ts^* at 18°C to avoid developmental effects. Approximately 30 females per line were shifted to 29°C to induce transgene expression. Flies were transferred to fresh food every 2 d, and dead flies were counted daily and discarded later.

### Statistical analysis

For each experiment, “*n*” represents the number of biological replicate, and error bar represents SEM. All experiments were repeated independently 3 times. Statistical significance was calculated by either unpaired *t*-test or 1-way ANOVA followed by the Tukey post hoc test if multiple comparisons were necessary using the GraphPad Prism Software. A log-rank test on the Kaplan–Meier data was utilized to determine the statistical significance in lifespan essay. Significance was accepted at the level of *P* < 0.05; * denotes *P* < 0.05; ** denotes *P* < 0.01; *** denotes *P* < 0.001; **** denotes *P* < 0.0001.

## Results

### Polo is specifically expressed in progenitor cells and shows a dynamic distribution during mitosis

In order to analyze the dynamic location of Polo in the *Drosophila* midgut in detail, the cellular localization of Polo was visualized using a protein trap strategy with GFP-tagged Polo (Polo-GFP) (Moutinho-Santos et al. 1999). Polo-GFP protein was significantly upregulated during mitosis in embryos (Kachaner et al. 2017). Since ISCs are largely quiescent during homeostatic conditions, *Drosophila* was orally infected with the entomopathogen *Ecc15*, which damaged the intestinal epithelium and induced compensatory proliferation of ISCs (Buchon et al. 2009; Lei et al. 2022). ISCs were identified based on the expression of *Dl-LacZ*, an enhancer trap of Dl (Delta) (Jiang et al. 2009). Moreover, immunostaining of the midgut was performed using an antibody against phosphorylated Histone H3 (PH3) to identify cells at different stages of mitosis (Li et al. 2012a).

At interphase, Polo-GFP showed a mostly diffuse cytoplasmic location, while it appeared as a single punctate (arrowhead) (Fig. 1c, I). During the transition from prophase to prometaphase, Polo-GFP accumulated rapidly within the nucleus (Fig. 1c, II and III). During metaphase and anaphase, Polo-GFP localized at the spindle poles and spindle microtubules (Fig. 1c, IV and V). At telophase, Polo-GFP was concentrated in the central spindle (Fig. 1c, VI). These results were consistent with a previous study reporting the expression of Polo-GFP during early embryogenesis in *Drosophila* (Moutinho-Santos et al. 1999). In addition, we also detected whether Polo was expressed in other cell types. EB cells (expressing *Su(H)GBE-LacZ*) (Furriols and Bray 2000) retained a weak Polo signal; no Polo signal was detected in ECs and EEs marked by the antibody against *nubbin* (Pdm1), a POU-domain transcription factor expressed specifically in mature ECs (Lee et al. 2009; Ma et al. 2019) and EE marker Prospero, respectively (Fig. 1d). These results suggested that Polo was specifically expressed in the progenitor cells and showed a dynamic distribution during mitosis.

### *Polo* depletion causes the loss of ISCs

**Fig. 2.**
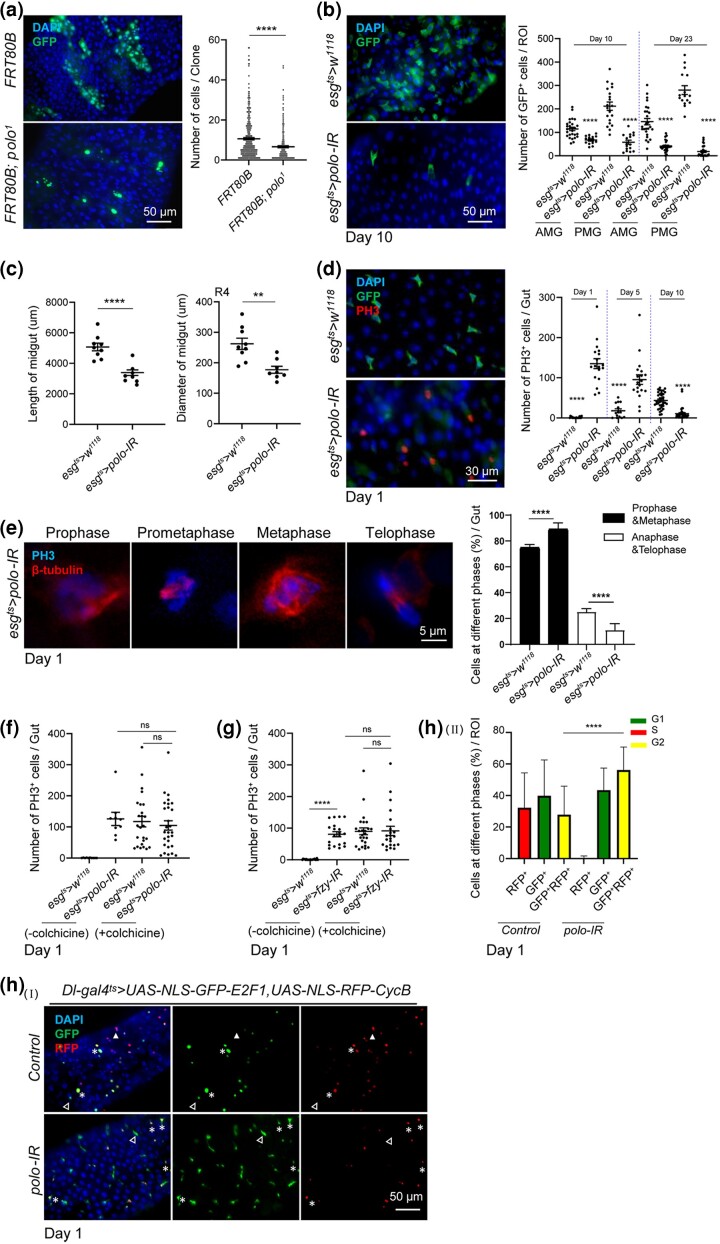
*Polo* depletion caused the loss of ISCs. a) Representative images of GFP-labeled MARCM clones of the R4 region of midguts with the indicated genotypes maintained at 25°C for 7 d after heat shock at 37°C for 30 min. The number of cells per clone was quantified. Scale bar, 50 *µ*m. b) Representative images of R4 region of midguts with the indicated genotypes maintained at 29°C for 10 d. The number of GFP^+^ cells per region of interest (ROI) in the anterior and posterior midguts was counted for statistical analysis on days 10 and 23, respectively. Scale bar, 50 *µ*m. c) Adult flies with the indicated genotypes were dissected for measuring the length and width of R4 region of midguts after being maintained at 29°C for 10 d. d) Representative images of the midguts with the indicated genotypes maintained at 29°C for 1 d. Antibody against PH3 was used for immunostaining. The number of PH3^+^ cells per midgut was quantified for different days. e) Representative images of the intestinal cells at different phases of mitosis after *polo* depletion in the progenitor cells maintained at 29°C for 1 d. Samples were stained for PH3 and β-tubulin, respectively. The ratio of cells at different phases to all PH3^+^ cells per gut was quantified. Scale bar, 5 *µ*m. f and g) Depletion of *polo* and *fzy* blocked dividing cells during mitosis. Adult flies with the indicated genotypes were orally fed with colchicine suspended in 5% sucrose or 5% sucrose alone and maintained at 29°C for 1 d. The number of PH3^+^ cells per gut was counted. h) Cell cycle was arrested at G2/M after *polo* depletion. The Fly-FUCCI system was applied for the analysis of the cell cycle in the midguts with the indicated genotypes. The GFP^+^, RFP^+^, and GFP^+^RFP^+^ indicated G1, S, and G2 phases of the cell cycle, respectively. Filled arrows showed GFP^-^RFP^+^ cells, empty arrows showed GFP^+^RFP^−^ cells, and asterisks showed GFP^+^RFP^+^ cells. (Ⅰ) Representative images of midguts with the indicated genotypes maintained at 29°C for 1 d. (Ⅱ) The relative proportions of GFP^+^ (G1), RFP^+^ (S), and GFP^+^RFP^+^ (G2) cells per ROI were quantified for statistical analysis. Means and SEMs (*n* = 20; Student's *t*-test). **P < 0.01; ***P < 0.001. Scale bar, 50 *µ*m.

In order to investigate the function of Polo in the midgut of adult *Drosophila*, the clones homozygous for *Polo* using the null allele *polo^1^* that cannot be phosphorylated (Tavares et al. 1996) were generated using the MARCM technique (Lee and Luo 2001). A reduction in the number of cells per clone as compared with the wild-type clones indicated that Polo was required for the ISC division (Fig. 2a). Furthermore, the Polo was inactivated, specifically in the progenitor cells, using *esg-Gal4 UAS-GFP tub-Gal80^ts^* system (hereafter referred to as *esg^ts^*) (Micchelli and Perrimon 2006). The flies, which were kept at 29°C to induce *polo* RNAi for 10 and 23 d with *esg^ts^*, showed a dramatic decrease in the number of GFP-positive (GFP^+^) cells in both the anterior and posterior midgut (Fig. 2b). Moreover, the lengths and widths in R4 region of posterior midguts also reduced as compared to the controls (Fig. 2c). In addition, the number of ISCs (as indicated by *Dl-LacZ*) also decreased significantly (Supplementary Fig. 1a). Consistent with the ISC/EB-specific expression pattern, the mRNA expression levels of *polo* were significantly reduced at different time points upon suppressing *polo* with the *esg^ts^* driver (Supplementary Fig. 1b).

The loss of ISCs is usually correlated with the abrogation of cell proliferation reflected by the reduction of mitotic marker PH3. By suppressing *polo* for 10 d, consistent with the loss of *esg > GFP^+^* progenitors, the number of PH3-positive (PH3^+^) cells indeed decreased significantly. However, surprisingly, an obvious increase was detected in the PH3^+^ cells on days 1 and 5 after initiating the knockdown experiment (Fig. 2d and Supplementary Fig. 1c). Additionally, the *polo* gene was suppressed specifically in EBs using *Su(H)GBE-Gal4 UAS-GFP tub-Gal80^ts^* (*GBE^ts^)* (Zeng et al. 2010) and in ISCs using *esg-Gal4 UAS-eYFP Su(H)-Gal80 tub-Gal80^ts^* (*ISC^ts^*) (Wang et al. 2014). The number of PH3^+^ cells increased significantly only with the ISC-specific driver at 29°C for 1 d (Supplementary Fig. 1d). *polo^9^* and *polo^10^* could arrest the cell cycle of stem cells in the larval brain and *Drosophila* S2 cells at metaphase (Donaldson et al. 2001; Bettencourt-Dias et al. 2004), a stage when cells were marked by PH3^+^ (Gurley et al. 1978; Li et al. 2012a, 2012b). These findings suggested that the PH3^+^ cells in the midgut on days 1 and 5 after *polo* depletion were arrested in mitosis. This hypothesis was supported by 3 pieces of evidence. First, the PH3^+^ cells at the anaphase and telophase stages were significantly less detected in the midguts with *polo* depletion in the progenitor cells than the controls. In contrast, the cells at prophase and metaphase were more easily detected in *polo*-depleted midguts (Fig. 2e). Second, the ingestion of colchicine, a drug used to arrest cells in the metaphase stage by preventing the polymerization of tubulin (Stapczynski et al. 1981), increased the number of PH3^+^ cells to a comparable level in *esg^ts^ > polo-RNAi* guts (Fig. 2f). In *esg^ts^ > polo-RNAi* midguts, the number of PH3^+^ cells did not further increase after feeding colchicine, consistent with the notion that the cell cycle of *polo*-depleted ISCs was already arrested around the prophase/metaphase stage. Finally, the fly-FUCCI system (fluorescent ubiquitination based on cell cycle indicator) (Zielke et al. 2014; Jin et al. 2020) was used to reveal the cell cycle status of *polo*-deficient ISCs. The results indicated an extended G2/M phase in the *polo*-depleted midgut (Fig. 2h). These data suggested that *polo*-depleted ISCs were arrested in prophase/metaphase and were subsequently lost.

### *Polo* depletion leads to aneuploidy and causes stem cell loss by premature differentiation

**Fig. 3.**
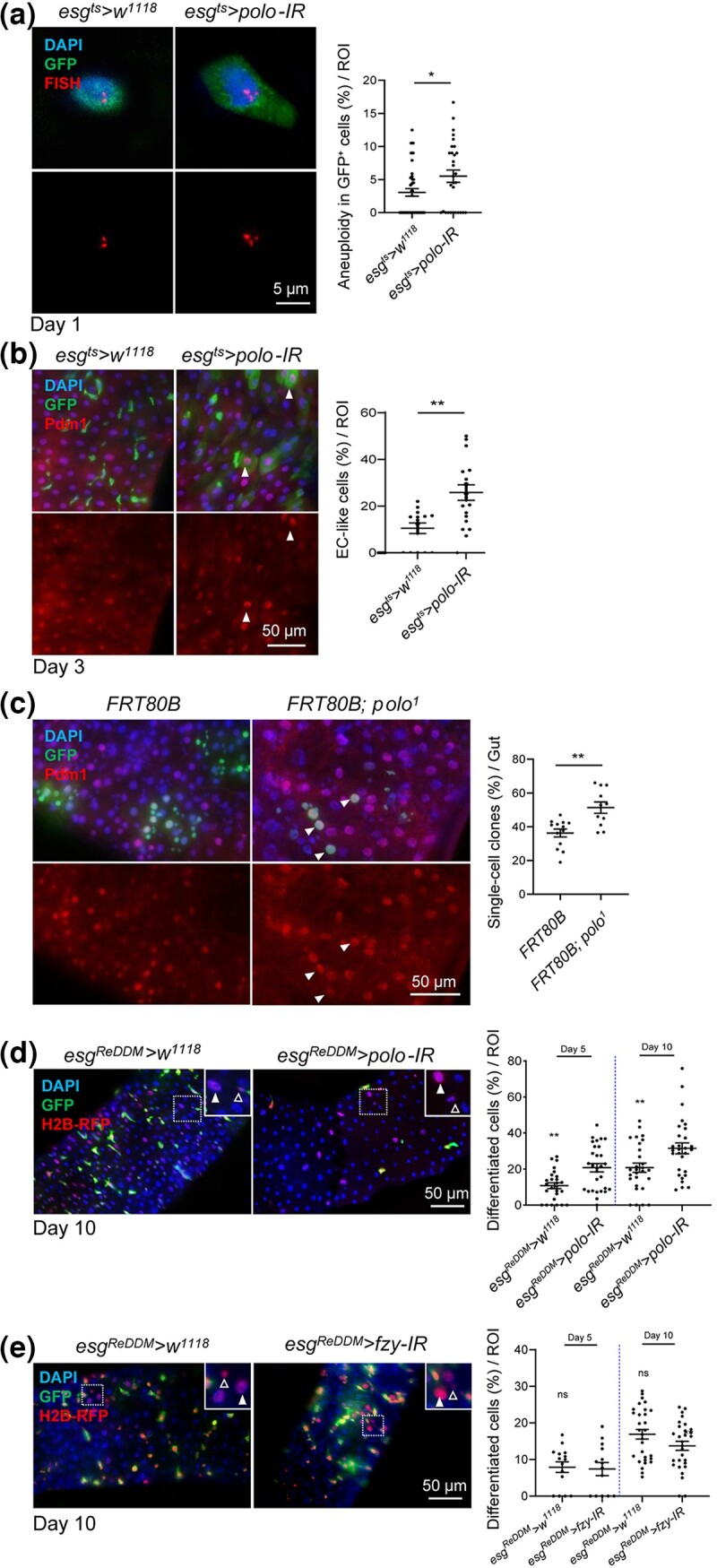
*Polo* depletion caused aneuploidy and premature differentiation in ISCs. a) FISH analysis in combination with immunofluorescence labeling chromosomes III with ISCs/EBs (GFP^+^DAPI^+^). Due to somatic chromosome pairing, the cells were only scored as aneuploid when more than 2 FISH signals were observed. The percentage of aneuploid cells in GFP-labeled cells per ROI was quantified. b) Premature differentiation after *polo* depletion. The antibody against EC marker Pdm1 was applied for immunostaining. The arrows indicate EC-like cells (Pdm1^+^GFP^+^). The ratio of newly formed EC-like cells to all the GFP^+^ cells in R4 region of control and *polo* RNAi midguts maintained at 29°C for 3 d was quantified. Scale bar, 50 *µ*m. c) Representative intestinal images of wild-type or *polo^1^* GFP^+^ MARCM clones maintained at 25°C for 7 d after heat shock at 37°C for 30 min. EC marker Pdm1 is shown in red. The arrows indicate single-cell clones stained positive for Pdm1. The percentage of single-cell clones in the wild-type and *polo^1^* per gut is shown. Scale bar, 50 *µ*m. d and e) Premature differentiation was confirmed by the *esg^ReDDM^* tracing system when *polo* knockdown rather than *fzy*. The representative intestinal images with the indicated genotypes were maintained at 29°C for 10 d. ISCs/EBs were double positive for GFP and H2B-RFP. Newly generated EEs (empty arrowhead) or polyploid ECs (filled arrowhead) were only labeled with H2B-RFP. The proportion of differentiated cells (GFP^−^RFP^+^ cells/all RFP^+^ cells) per ROI was quantified on days 5 and 10, respectively. Means and SEMs (*n* = 30; Student's *t*-test). **P < 0.01; ***P < 0.001. Scale bar, 50 *µ*m.

Next, we checked the mechanism by which *polo*-depleted G2/M-arrested ISCs were lost. Polo plays a crucial role in regulating the SAC pathway to maintain genomic stability during mitosis (Conde et al. 2013a, 2013b; Cordeiro et al. 2020). Previous studies showed in different model organisms and cell types that a defective SAC induces aneuploidy (Resende et al. 2018; Mikwar et al. 2020). We thus investigated if *polo*-depletion in ISCs induces aneuploidy. The number of third chromosomes per cell was detected using FISH (Gogendeau et al. 2015; Resende et al. 2018). The results showed a significant increase in the proportion of ISCs carrying aneuploidy in the *polo*-depleted midguts as compared with controls (Fig. 3a). Therefore, depletion of *polo* induced aneuploidy in ISCs.

The aneuploid cells usually undergo apoptosis (Thompson and Compton 2010; Sabino et al. 2015). Therefore, it was initially suspected that *polo*-depleted ISCs were lost by apoptosis. Recent studies show that caspase inhibitor *Diap1* (Orme and Meier 2009) signal was weekly detected in ISCs, in contrast, was strong in EBs, which indicated that classical programmed cell death (PCD) seemed to affect EB rather than ISC (Reiff et al. 2019). Therefore, we used *esg^ts^* (ISCs/EBs) driver to detect apoptotic progenitor cells. The number of progenitor cells being positive for caspase-3 in the midgut was always very low as dying cells were cleared off rapidly from the intestinal epithelium, which hampered quantification (Reiff et al. 2019). Therefore, we performed TUNEL (TdT-mediated dUTP Nick-End Labeling) assay in addition to immunostaining with an antibody detecting cleaved caspase-3, and no apoptotic progenitor cells were detected (Supplementary Fig. 2a and b). Coexpression of *p35*, a baculoviral caspase inhibitor (Hay et al. 1994), did not prevent the loss of progenitors with *polo* depletion (Supplementary Fig. 2c).

Studies have shown that aneuploidy in ISCs has contrasting effects, either causing ISC overproliferation to form gut dysplasia (Resende et al. 2018; Brás et al. 2021) or ISC loss due to premature differentiation (Gogendeau et al. 2015). Our results were apparently against the notion that aneuploidy increased ISC proliferation. The effects of *polo* depletion in causing stem cell loss in the long term (examined at 10 and 23 d of *polo* RNAi) by inducing the differentiation of ISCs were further validated using the 3 methods as follows. First, *polo* was suppressed by RNAi using the *esg^ts^* driver at 29°C for only 3 d. The ratio of newly formed EC-like cells (GFP^+^Pdm1^+^) to all GFP^+^ cells in R4 region of *polo* RNAi midguts was significantly increased through staining with EC marker Pdm1, suggesting that these progenitor cells were undergoing rapid differentiation (Fig. 3b). Second, the clonal analysis using MARCM demonstrated that a high ratio of *polo^1^* clones was single-cell clones, which were stained positive for Pdm1 (Fig. 3c). Together with the earlier findings that the average number of cells decreased per clone in *polo^1^* (Fig. 2a), aneuploidy by *polo^1^* limited the ability of ISCs to produce progeny and promoted ISCs loss by becoming single EC clones. Third, the *esg^ReDDM^* (repressible dual differential stability cell marker) tracing technique (Antonello et al. 2015; Zipper et al. 2022) was used to reveal the fate of *polo*-depleted ISCs. In addition to labeling ISCs/EBs with a short-lived mCD8-GFP, this system also expressed a long-lived histone-tethered red fluorescent protein (H2B-RFP) in *esg-Gal4* progenitors, which propagated into the daughter cells, allowing the tracing of newly differentiated epithelial cells. The results showed that the differentiation index (calculated as the ratio of GFP^−^RFP^+^ cells/all RFP^+^ cells) increased after suppressing the *polo* gene for 5 and 10 d (Fig. 3d). In order to determine whether differentiation resulted from the general mitotic abnormality or was specific to *polo* depletion, we also depleted another mitotic gene *fzy*, which is required for anaphase progression in mitotic cells (Swan and Schüpbach 2007). This manipulation arrested cells at 1-cell stage, specifically at metaphase (Kitagawa et al. 2002; Stein et al. 2007). We found that *fzy*-depleted ISCs were arrested in mitosis (Fig. 2g and Supplementary Fig. 2d) rather than underwent premature differentiation (Fig. 3e). We therefore conclude that the premature differentiation was a direct consequence of *polo* depletion. In addition, there was no premature differentiation phenotype when *polo* was depleted in EBs (Supplementary Fig. 2e). Therefore, these findings suggested that the loss of Polo decreased ISCs by driving their premature differentiation into ECs, without inducing apoptosis or proliferation of stem cells.

### Enforced expression of active *polo^T182D^* causes the loss of ISCs in the midgut

**Fig. 4.**
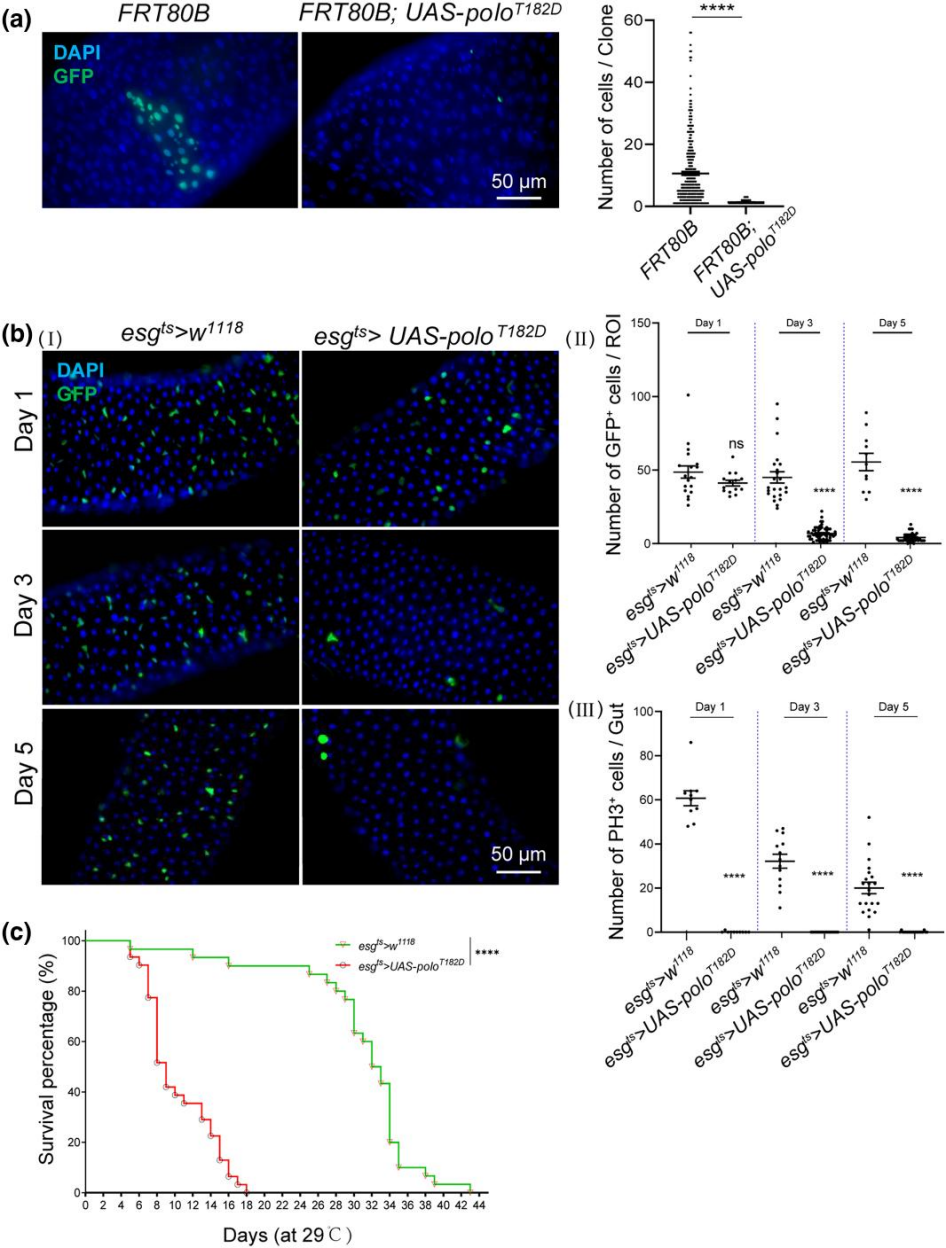
Enforced expression of active *polo^T182D^* caused the loss of ISCs in midguts. a) Representative images of GFP-labeled MARCM clones from flies with indicated genotypes maintained at 25°C for 7 d after heat shock at 37°C for 30 min. The number of cells per clone was counted. Scale bar, 50 *µ*m. b) (Ⅰ) Representative images in R4 region of posterior midguts with the indicated genotypes maintained at 29°C for different days. (Ⅱ) The number of GFP^+^ cells per ROI is shown. (Ⅲ) At each indicated time point, *Ecc15* was fed to induce cell division for another 24 h. The number of PH3^+^ cells per gut was quantified. Means and SEMs (*n* = 25; Student's *t*-test). **P < 0.01; ***P < 0.001. c) Survival assay after the overexpression of *polo^T182D^* in progenitor cells. Adult flies were maintained at 29°C. The analysis of Mantel-cox data showed a statistically significant difference in survival rates between the control and *polo^T182D^* overexpressing *Drosophila*.

Next, the effects of elevated Polo activity in the ISCs were investigated by overexpressing a constitutively active Polo (*polo^T182D^*) (Carmena et al. 2012; Khaminets et al. 2020). MARCM technique was used to express the *polo^T182D^* gene in clones positively marked with GFP. The wild-type clones grew normally and contained various cells, while the clones with *polo^T182D^* overexpression were largely single-cell clones (Fig. 4a). The *esg^ts^* system, driving *polo^T182D^* expression at 29°C for different days, showed a dramatic decrease in the number of GFP^+^ cells (Fig. 4b, I and II). Moreover, *polo^T182D^* overexpression with *esg^ts^* inhibited proliferation of ISCs both in homeostatic (Supplementary Fig. 3a) and infected conditions (infection with *Ecc15*; Fig. 4b, III), suggesting a reduction in the functional ISCs. Not surprisingly, the prolonged expression of *polo^T182D^* greatly reduced the lifespan of *Drosophila* (Fig. 4c). In addition, to further delineate the role of Polo in the proliferation of ISCs, *polo^T182D^* was either specifically overexpressed in EBs using *GBE^ts^* or in ISCs using *ISC^ts^*. The results indicated that only the ISC-specific activation of Polo restricted the proliferation of stem cells (Supplementary Fig. 3b). In contrast to the strong effects of *polo^T182D^*, overexpressing the wild-type *polo* with *esg^ts^* showed no effects on stem cell division (Supplementary Fig. 3c). These results were consistent with the fact that Polo itself has to be phosphorylated prior to showing its kinase activity (Tavares et al. 1996; Carmena et al. 2012). In summary, the constitutive Polo activity in ISCs caused their loss.

### Overexpression of *polo^T182D^* induces the abnormal accumulation of β-tubulin and drives ISCs apoptosis

**Fig. 5.**
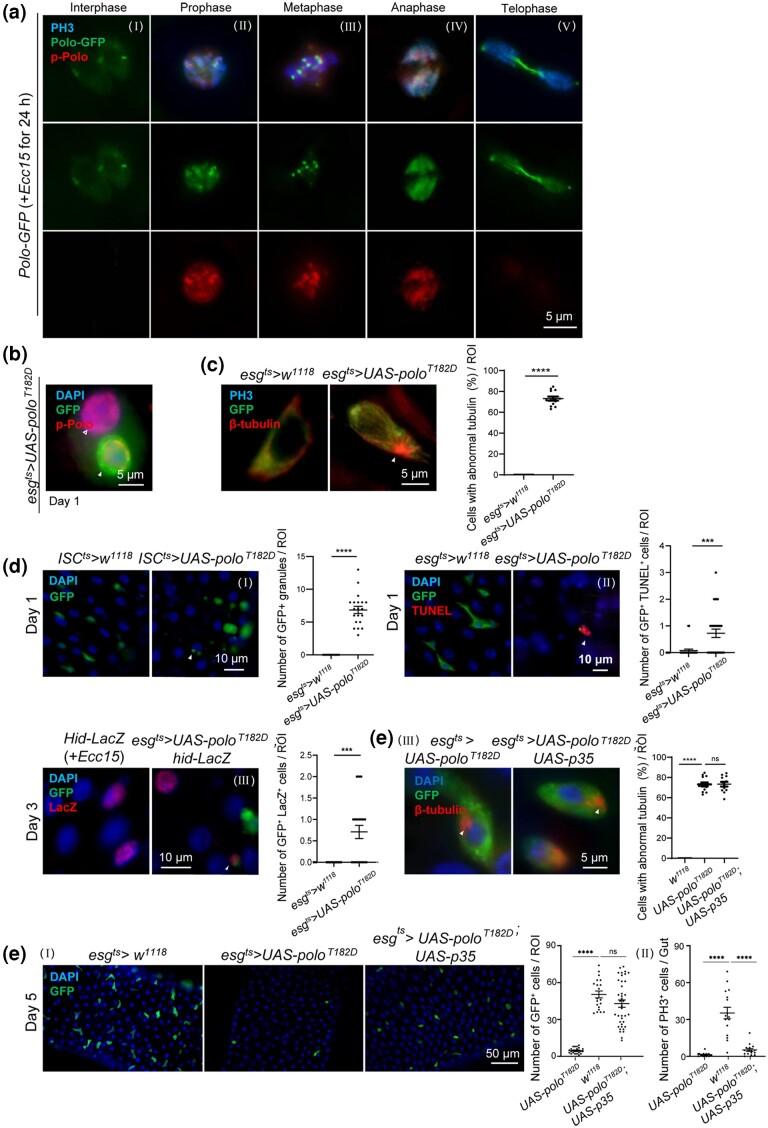
Enforced *polo^T182D^* induced the abnormal accumulation of β-tubulin and drove progenitor apoptosis. a) Active Polo protein entered nucleus and showed a dynamic localization at different phases of mitosis in the dividing cells. Immunostaining was used to label PH3 and pPolo. Adult flies with Polo-GFP were orally infected with *Ecc15* to induce the ISC division for 24 h. Scale bar, 5 *µ*m. b) Representative images of progenitor cells with *polo^T182D^* overexpression at 29°C for 1 d. Antibody against pPolo was used for immunostaining. pPolo was detected inside the nucleus of progenitor cells with weak (empty arrowhead) or strong (filled arrowhead) GFP fluorescence. Scale bar, 5 *µ*m. c) Representative images of cells in the control and *polo^T182D^* overexpressing midguts, flies with *Ecc15* ingestion at 29°C for 10 h followed by maintaining at 18°C for 12 h. Antibodies against β-tubulin and PH3 were applied for immunostaining. The arrow indicates the abnormal associated β-tubulin. The ratio of cells with abnormal tubulin to all GFP^+^ cells per ROI was quantified. Scale bar, 5 *µ*m. d) Overexpression of *polo^T182D^* in progenitor cells induced ISCs apoptosis. (Ⅰ) Apoptotic granules were detected in *UAS-polo^T182D^ Drosophila* with *ISC^ts^* maintained at 29°C for 1 d. (Ⅱ) TUNEL^+^ cells were detected in the midguts of *Drosophila* with *UAS-polo^T182D^* overexpression at 29°C for 1 d. (Ⅲ) LacZ^+^ cells were found in *UAS-polo^T182D^ Drosophila*, which was induced at 29°C for 3 d. Similar cells were also detected in the control *Drosophila* orally infected with *Ecc15*. The number of GFP^+^ granules, GFP^+^TUNEL^+^ cells, and GFP^+^ LacZ^+^ cells per ROI was counted. The arrows indicate apoptotic ISCs. Means and SEMs (*n* = 20; Student's *t*-test). Scale bar, 10 *µ*m. e) Coexpression of *p35* rescued the number but not the activity and abnormal tubulin of ISCs disrupted by the overexpression of *polo^T182D^*. (Ⅰ) The representative images of R4 region of posterior midguts isolated from adults with the indicated genotypes maintained at 29°C for 5 d. The number of GFP^+^ cells per ROI was counted. Scale bar, 50 *µ*m. (Ⅱ) In another group, the *Drosophila* adults were kept at 29°C for 1 d followed by *Ecc15* ingestion for another day. The number of PH3^+^ cells per gut was quantified. (Ⅲ) Representative images of cells in the midguts with the indicated genotypes. Flies were orally infected with *Ecc15* at 29°C for 10 h followed by maintaining at 18°C for 12 h. Antibody against β-tubulin was used for immunostaining. The arrows indicate the abnormal accumulation of β-tubulin. The ratio of cells with abnormal tubulin to all GFP^+^ cells per ROI was quantified. Means and SEMs (*n* = 25; 1-way ANOVA). **P < 0.01; ***P < 0.001. Scale bar, 5 *µ*m.

In order to understand whether *polo^T182D^* could drive the loss of stem cells in the same manner as that of *polo* depletion, the localization of active Polo was first analyzed. The phosphorylation of the conserved Thr210 can activate Polo ortholog Plk1 (Macůrek et al. 2008). The phosphorylated Polo (pPolo) was detected using the antibody against phosphorylated Plk1 (phospho T210) in cells (Carmena et al. 2012). The oral infection of *Ecc15* was again used to stimulate ISC proliferation. pPolo was not detected in cells at the interphase stage when Polo-GFP showed a mostly diffuse cytoplasmic location (Fig. 5a, I); however, after prophase onset, Polo-GFP entered nucleus and pPolo colocalized with it (Fig. 5a, II to Ⅳ). At telophase, Polo-GFP was concentrated in the central spindle, while pPolo gradually disappeared (Fig. 5a, V). These results were consistent with a previous study, which reported that phosphorylation of Polo triggered its entry into the nucleus (Kachaner et al. 2017)

As expected, pPolo relocated to the nucleus after the overexpression of *polo^T182D^* (Fig. 5b). This manipulation was correlated with the abnormal accumulation of β-tubulin, which clumped to 1 specific side of the cytoplasm (Fig. 5c). After 24 h of *polo^T182D^* overexpression, the GFP^+^ granules reminiscent of apoptotic cells were observed (Fig. 5d, I). The TUNEL to detect the apoptotic progenitor cells after *polo^T182D^* overexpression suggested that these cells underwent apoptosis (Fig. 5d, II). This result was further supported by the expression of *hid-LacZ*, a reporter indicating the expression of a proapoptotic gene *head involution defective* (*hid*) (Ma et al. 2012) (Fig. 5d, III). Furthermore, the simultaneous expression of a caspase inhibitor *p35* suppressed the loss of stem cells caused by *polo^T182D^* (Fig. 5e, I) but did not rescue their activity and the abnormal accumulation of β-tubulin in cells (Fig. 5e, II and III). In the meanwhile, *esg^ReDDM^* was used to reveal the fate of ISCs with *polo^T182D^* overexpression, and differentiated progeny was not observed (Supplementary Fig. 3d). Altogether, these data demonstrated that the continuous expression of active *polo^T182D^* led to the abnormal accumulation of β-tubulin and the apoptosis of ISCs.

### *Polo* is a direct target gene of Sox21a

**Fig. 6.**
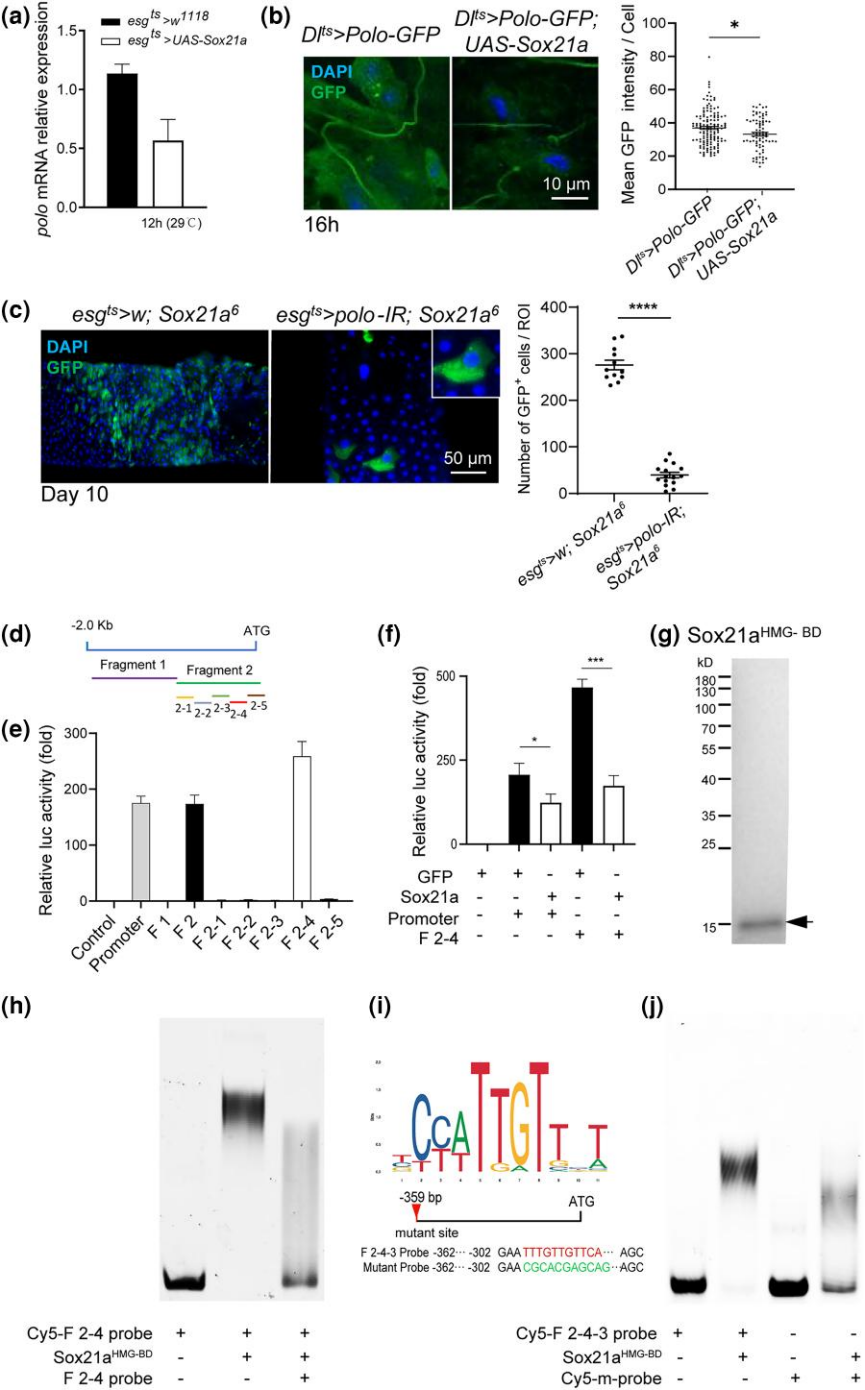
*Polo* was a direct target gene of Sox21a. a) qPCR quantification of *polo* mRNA expression levels in control and adult *Drosophila* received *Sox21a* overexpression in the progenitor cells maintained at 29°C for 12 h. Normalized results are presented relative to normalized expression levels in control (*esg^ts^ > w^1118^*), which was set as 1. b) Overexpression of *Sox21a* inhibited the expression of Polo. Confocal images of R4 region of posterior midguts were isolated from adult flies with the indicated genotypes maintained at 29°C for 16 h. GFP fluorescence intensities in GFP^+^ cells were quantified for statistical analysis. Means and SEMs (*n* = 40; Student's *t*-test). Scale bar, 10 *µ*m. c) Representative images of midguts with the indicated genotypes maintained at 29°C for 10 d. The number of GFP^+^ cells per ROI was quantified. Scale bar, 50 µm. d) A 2-kb upstream DNA sequence of the *polo* gene transcription start site was divided into several overlapping sequences to create different pGL3-Polo reporter vectors. e) Dual-Luciferase assay to detect the activation of *polo* promoter. *Drosophila* S2 cells were cotransfected with the pGL3-Polo reporter vectors and the internal control Copia–Renilla vector. After 48 h, luciferase activity was detected in each cell lysate (*n* = 3 times). f) S2 cells were transfected with the luciferase reporters driven by *polo* promoter, F2-4, and internal control Copia–Renilla vector, along with pAc-GFP as control or pAc-Sox21a plasmid to overexpress Sox21a. After 48 h, luciferase activity was detected in each cell lysate (*n* = 3 times). g) Purification of Sox21a^HMG-BD^. The 108–208 aa fragment of Sox21a was cloned and expressed in *Escherichia coli* for protein purification. The arrow indicates purified Sox21a^HMG-BD^. h) EMSA of Sox21a^HMG-BD^ protein bound to the F2-4 region as described in e) of *polo* promoter. i) Predicted possible binding site of F2-4-3 to Sox21a with online database at Jaspar. Schematic representation of F2-4-3 probe containing the wild-type sequence and the probe containing mutated sequence. j) EMSA of Sox21a^HMG-BD^ protein bound to the F2-4-3 wild-type probe but not the mutated probe.

*Sox21a*, a *Drosophila* B group Sox gene, is specifically expressed in intestinal progenitor cells and plays an important role in regulating the proliferation and differentiation of ISCs (Zhai et al. 2015, 2017). The role of Polo in the maintenance and differentiation of ISCs urged to test the effects of *Sox21a* on the *polo* expression. First, the overexpression of *Sox21a*, which primed the differentiation of stem cells, decreased the expression of *polo*, detected using quantitative PCR (qPCR) (Fig. 6a). Further quantification of the Polo-GFP intensity using *Dl >* GFP to indicate ISCs confirmed that the Polo protein levels were reduced when *Sox21a* gene was overexpressed in ISCs (Fig. 6b). In addition, loss of *Sox21a* blocked the differentiation program of EBs, which resulted in EB accumulation and formation of tumors (Zhai et al. 2015). The EB accumulation due to loss of *Sox21a* was surprisingly suppressed by *polo* depletion-induced premature differentiation (see Discussion section) (Fig. 6c).

A previous study using the DamID technique to identify Sox21a targets identified *polo* (Doupé et al. 2018). In order to validate that Sox21a directly regulates *polo*, the Dual-Luciferase system (Xu et al. 2013; Pan et al. 2022) was used to identify the activation of *polo* promoter in *Drosophila* S2 cells. A 2-kb DNA sequence upstream of the *polo* gene transcription start site was divided into fragments with a 50-bp overlap between each adjacent fragment as indicated (Fig. 6d). Then, firefly luciferase reporters driven by these fragments were generated and introduced into the S2 cells. This led us to find a fragment (referred as to F2-4) that exhibited a significant luciferase expression (Fig. 6e). Dual-Luciferase system was used again to verify the relationship between Sox21a and F2-4 in *Drosophila* S2 cells. Luciferase activity controlled by F2-4 showed a significant decrease upon Sox21a expression (Fig. 6f). Then, the HMG-BD, a DNA-binding domain of Sox21a (Sox21a^HMG-BD^), was purified (Fig. 6g), and the interactions between the recombinant Sox21a^HMG-BD^ and Cy5-labeled probe (F2-4) were tested using EMSA (Fried 1989; Ma et al. 2020). The DNA–protein complex showed a slower migration speed as compared with the free DNA, indicating a direct interaction of Sox21a^HMG-BD^ with the labeled DNA (Fig. 6h). In addition, competition assays confirmed the binding by performing EMSA again by adding simultaneously excess unlabeled probe (Fig. 6h). To reveal the putative binding sites for HMG domain of Sox transcription factors on the F2-4, Dual-Luciferase system was used again to identify a sub-fragment of F2-4, the F2-4-3, which exhibited a significant luciferase expression (Supplementary Fig. 4). With the Jaspar database to predict Sox-binding sites (https://jaspar.genereg.net), TTTGTTGTTCA was established as a candidate (Fig. 6i). The gel-shift assay showed that the Sox21a^HMG-BD^ interacted with the probe containing the wild-type sequence but not the probe containing mutated sequence (Fig. 6j), thus confirming a specific interaction between the Sox21a proteins with the promoter of *polo*. Therefore, these results supported the hypothesis that *polo* was a transcriptional target gene of Sox21a.

## Discussion

**Fig. 7.**
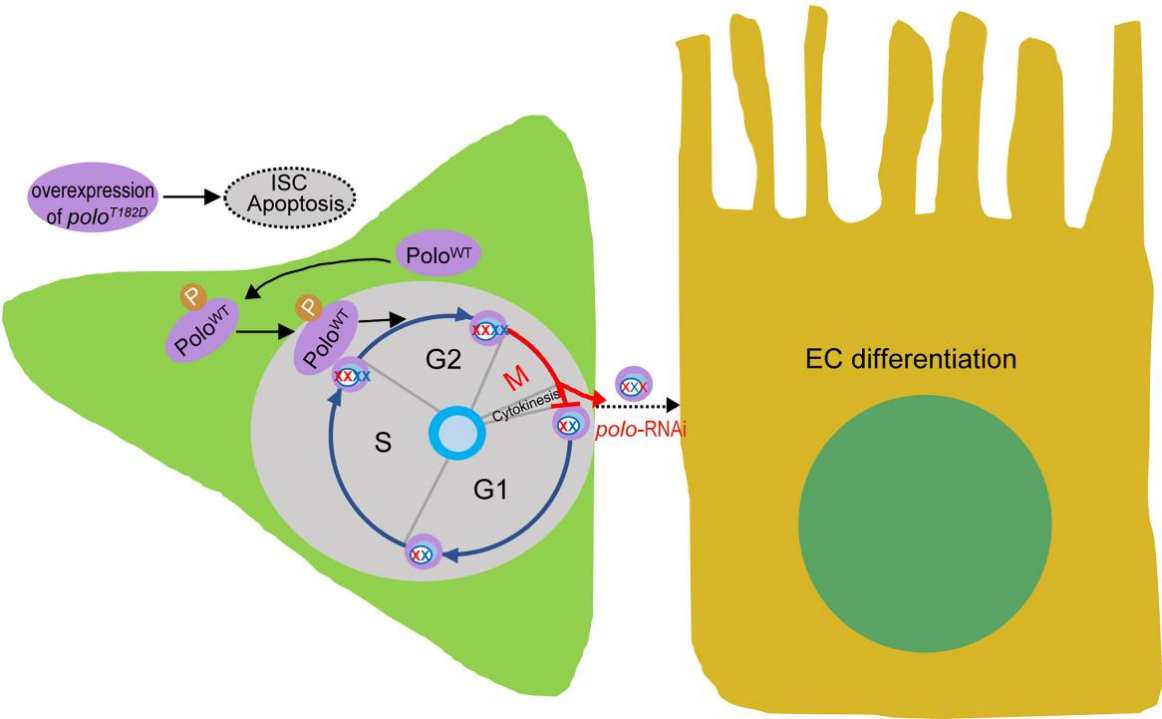
Polo activity should be properly maintained for optimal stem cell function. Phosphorylated wild-type Polo (Polo^WT^) translocated to the nucleus and promoted the process of mitosis. The ISCs with *polo*-suppression, induced by either gene interference or gene mutation, were correlated with an extended G2/M phase and aneuploidy and were subsequently lost by premature differentiation into ECs. In contrast, overexpression of the constitutively active Polo (*polo^T182D^*) significantly enhanced Polo activity, which suppressed ISC proliferation and caused ISC loss via apoptosis. XX, XXX, and XXXX refer to diploid, aneuploid, and polyploidy genomes, respectively.

The current study reported a specific function of Polo-mediated mitosis in regulating the stem cells in *Drosophila* midgut. The results indicated that the Polo activity should be properly maintained for optimal stem cell function (Fig. 7). The results also revealed that the depletion of *polo* could cause a reduction in the gut size due to a gradual decrease in the number of functional ISCs. The *polo*-deficient ISCs were correlated with an extended G2/M phase and aneuploidy and were subsequently lost by premature differentiation into ECs. In contrast, the constitutively active Polo (*polo^T182D^*) suppressed ISC proliferation and drove the ISC loss via apoptosis.

The finding that EB accumulation caused by *Sox21a* mutation was rescued by *polo* depletion (Fig. 6c) was consistent with a previous study showing *polo* was a direct target gene of Sox21a through DamID technique (Doupé et al. 2018). However, since Polo plays a key role in mitosis, we believe that the reason for the rescue is due to the aneuploid effect rather than Polo acting in the genetic network controlling normal differentiation. This underlies an interesting hypothesis that ISCs with impaired mitosis by default undergo rapid differentiation regardless of the presence of an intact genetic cascade required for normal differentiation. Such “safe-guarding” mechanism seems deeply rooted in the design of ISCs and should enable elimination of abnormal stem cells, representing a fitness selection process to maintain tissue health. Depletion of both *polo* and *fzy* arrested ISCs in mitosis, but the fact that only *polo*-depleted ISCs underwent premature differentiation supports the abnormal differentiation was a direct consequence of *polo* depletion. However, it is currently not clear how *polo*-depletion resulted in stem cell loss by differentiation into ECs, which may involve a shift in the mode of normally asymmetric stem cell division (O’Brien et al. 2011).

SAC, a surveillance mechanism, preventing or delaying the mitotic exit in response to abnormal interaction between chromosomes and mitotic spindle, is important for maintaining genomic stability (Musacchio and Salmon 2007; Lara-Gonzalez et al. 2021). There are contrasting results about the ISCs response to aneuploidy that occurred by the depletion of SAC proteins: the depletion of bub3 caused the loss of ISC by differentiation (Gogendeau et al. 2015), while the depletion of BubR1, mad2, or mps1 could increase the proliferation of ISCs and accumulation of ISCs/EBs and EEs in the *Drosophila* midgut (Resende et al. 2018). Since Polo is a mitotic kinase proposed to be involved in SAC function (Conde et al. 2013a, 2013b; Cordeiro et al. 2020), similar to the effect of knocking down SAC protein bub3 in ISCs, *polo*-deficient ISCs were correlated with aneuploidy and were subsequently lost by premature differentiation into ECs.

A major feature of cancer cells is hyperproliferation; blocking cell division can be used as a strategy for the treatment of tumors. Colchicine, an antimitotic agent, could inhibit mitosis by preventing the polymerization of tubulin (Stapczynski et al. 1981). Targeting tubulin–colchicine site was applied for cancer therapy (Duan et al. 2019) and the administration of colchicine resulted in tumor loss in the *Drosophila* gut (Markstein et al. 2014). In a mouse model, colchicine suppressed tumor growth by inducing cell apoptosis (Cho et al. 2017; Yan et al. 2020). Plk1 plays an important role in the multiple aspects of the cell cycle and is overexpressed in tumors, thereby making the Plk1 an attractive target for cancer therapeutics (Iliaki et al. 2021; Kressin et al. 2021; Zhang et al. 2021). Plk1 was considered as an oncogene (Smith et al. 1997; Takai et al. 2005; Gheghiani et al. 2021) but now is under debate (de Cárcer 2019; Iliaki et al. 2021). Notch deficiency in progenitor cells led to the overproliferation of ISCs and the formation of tumors, which were mainly composed of ISCs and EEs in *Drosophila* (Ohlstein and Spradling 2006; Patel et al. 2015). The current study observed that the *polo* depletion did not result in the formation of tumor when *Notch* was knockdown in ISCs; this indicated that aneuploidy might decrease the tumorigenic potential of ISCs in the fly midgut. In addition, the constitutively active *polo^T182D^* suppressed ISC proliferation and drove the ISC loss via apoptosis. As our data of both Polo LOF and GOF pointed to a loss of stem cells albeit due to different mechanisms, the role of *polo* as an oncogene in *Drosophila* midgut remains unresolved. Recent studies reported that loss of function of Notch receptor is not frequently observed in colorectal cancer (CRC) tumorigenesis (Zipper et al. 2022). Therefore, future work using the *Drosophila* model of CRC based on CRISPR/Cas9-induced gene excision of *apc1*, *apc2* (*adenomatous polyposis coli 1 and 2*), *p53*, *Medea* (*dSmad4*), and *Pten* (*Phosphatase* and *tensin* homolog) (Bangi et al. 2016; Zipper et al. 2022) will be needed to answer the question.

This study demonstrated that the constitutively active *polo^T182D^* led to the abnormal accumulation of β-tubulin and induced the apoptosis of stem cells in *Drosophila* midgut. A previous study reported that cohesin controlled ISC identity, in which *polo^T182D^* showed differentiation phenotype (Khaminets et al. 2020). Moreover, after the overexpression of *polo^T182D^* with *esg^ReDDM^*, some RFP^+^GFP^−^ polyploid ECs were also detected. Since the overexpression of *polo^T182D^* did not affect EB (Supplementary Fig. 3b), it was not surprising to observe the differentiated cells in *esg^ReDDM^* flies, which was consistent with the previous studies. The enforced expression of active *polo^T182D^* has also been proposed to be toxic in other tissues, inhibiting the development of a majority of embryos before hatching or inducing smaller and rougher eyes (Kachaner et al. 2017). Altogether, this study concluded that Polo activity should be properly maintained for optimal stem cell function.

## Supplementary Material

jkad084_Supplementary_Data

## Data Availability

The authors affirm that all the data necessary for drawing the conclusions are present in the text, figures, and figure legends. Most of the *Drosophila* stocks are obtained from Bloomington or Vienna Stock center, with identifiers listed in the Materials and methods section. All the other lines are available upon request. Supplemental material available at G3 online.
