# Supplementary material for: Regulation of intestinal stem cell activity by a mitotic cell cycle regulator Polo in Drosophila
Source: G3 (Bethesda). 2023 Jun 1;13(6):jkad084. doi: 10.1093/g3journal/jkad084 (PMC10234410; doi:10.1093/g3journal/jkad084)
Supplement: jkad084_Supplementary_Data [file jkad084_supplementary_data.zip › Supplemental_Figure_Legends_G3-2023-404082.docx]

**Fig. S1.** *polo* depletion caused the loss of ISCs. a) Representative images of the midguts with the indicated genotypes maintained at 29℃ for 10 days. ISCs were marked with *Dl-LacZ*. The number of LacZ-positive cells per ROI was counted for statistical analysis. Means and SEMs (n=20; Student’s t test). Scale bar, 50 μm. b) qPCR quantification of *polo* expression levels in control and adults received *polo* depletion in progenitor cells at different time points. Normalized results are presented relative to normalized expression levels of control (*esg^ts^>w^1118^*), which was set as 1. c) Representative images of R4 region of midguts with the indicated genotypes maintained at 29℃ for 5 and 10 days. Antibody against PH3 was used for immunostaining. Scale bar, 50 μm. d) Depletion of *polo* in ISCs (*ISC^ts^)* but not in EBs (*GBE^ts^)* caused a significant increase in the number of PH3^+^ cells in *Drosophila* midguts. Representative images of the midguts with the indicated genotypes maintained at 29℃ for 1 day. The number of PH3^+^ cells per gut was counted for statistical analysis. Means and SEMs (n=25; Student’s t test). **p<0.01, ***p<0.001. Scale bar, 50 μm.

**Fig. S2.** Loss of ISCs caused by *polo* depletion was not due to apoptosis. a) Representative images of the midguts with the indicated genotypes maintained at 29℃ for 1 day. Immunostaining labeled the cleaved caspase-3. Scale bar, 50 μm. b) Representative images of the midguts with the indicated genotypes maintained at 29℃ for 2 days. TUNEL-positive cells were only detected in the control *Drosophila* orally infected with *Ecc15*. c) Representative images of midguts with the indicated genotypes maintained at 29℃ for 10 days. The number of GFP^+^ cells per ROI was counted for statistical analysis. Means and SEMs (n=25; one-way ANOVA). **p<0.01, ***p<0.001. d) Representative images of the midguts with the indicated genotypes maintained at 29℃ for 1 day. Antibody against PH3 was used for immunostaining. The number of PH3^+^ cells per midgut was quantified. Means and SEMs (n=20; Student’s t test). **p<0.01, ***p<0.001. e) Representative images in R4 region of midguts with the indicated genotypes maintained at 29℃ for 3 days. Immunostaining labeled Pdm1. Scale bar, 50 μm.

**Fig. S3.** Overexpression of active *polo^T182D^* caused the loss of ISCs. a) The number of PH3^+^ cells with the indicated genotypes maintained at 29℃ for different days was quantified in the absence of *Ecc15* infection. b) Overexpression of *polo^T182D^* in ISCs (*ISC^ts^*) but not in EBs (*GBE^ts^*) caused the loss of cells. Representative images of the midguts with the indicated genotypes maintained at 29℃ for 5 days. The number of GFP^+^ cells per ROI was counted for statistical analysis. c) Representative images of midguts with the indicated genotypes maintained at 29℃ for 1 day. Antibody against HA was used for immunostaining. The number of PH3^+^ cells per gut was counted for statistical analysis. d) The representative intestinal images of R4 region with the indicated genotypes maintained at 29℃ for 1 day and 5 days. ISCs/EBs were double positive for GFP and H2B-RFP. Newly generated EEs or polyploid ECs were only labeled with H2B-RFP. The proportion of differentiated cells (GFP^−^RFP^+^ cells / all RFP^+^ cells) per ROI was quantified on day 1 and day 5, respectively. Means and SEMs (n=30; Student’s t test). **p<0.01, ***p<0.001. Scale bar, 50 μm.

**Fig. S4.** Dual-Luciferase^®^ assay was used to detect the activation of F2-4. *Drosophila* S2 cells were co-transfected with the pGL3 reporter vectors driven by *polo* promoter fragments and the internal control Copia-Renilla vector. After 48 h, luciferase activity was detected in each cell lysate (n=3 times).
