# Supplementary figures and images for: Regulation of intestinal stem cell activity by a mitotic cell cycle regulator Polo in Drosophila
Source: G3 (Bethesda). 2023 Jun 1;13(6):jkad084. doi: 10.1093/g3journal/jkad084 (PMC10234410; doi:10.1093/g3journal/jkad084)

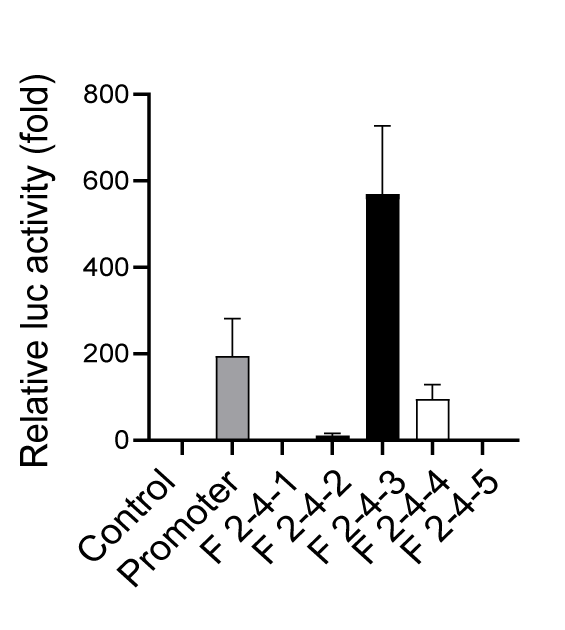

Supplement: jkad084_Supplementary_Data [file jkad084_supplementary_data.zip › Fig._S4_G3-2023-404082.tif]

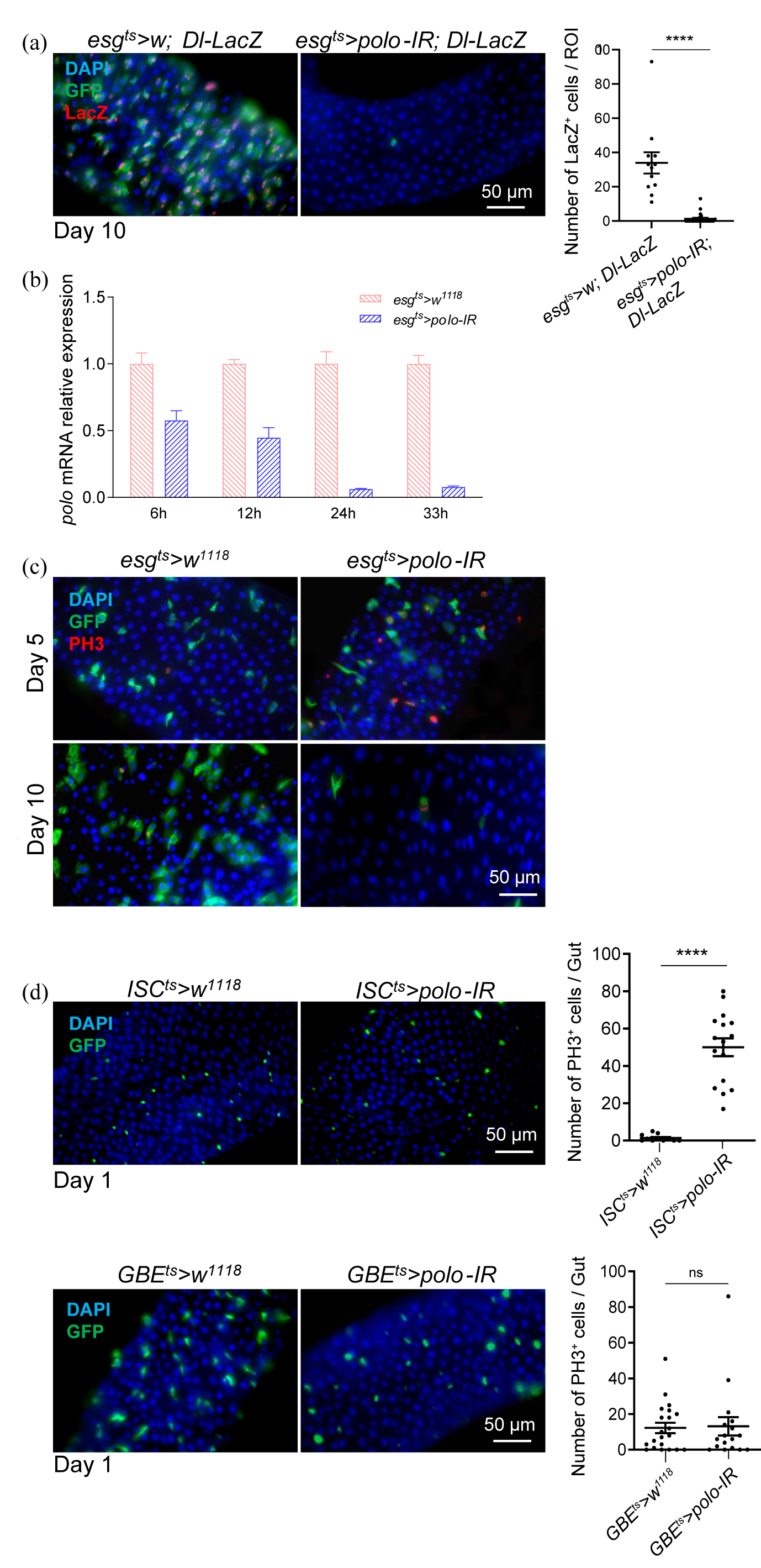

Supplement: jkad084_Supplementary_Data [file jkad084_supplementary_data.zip › Fig._S1_G3-2023-404082.tif]

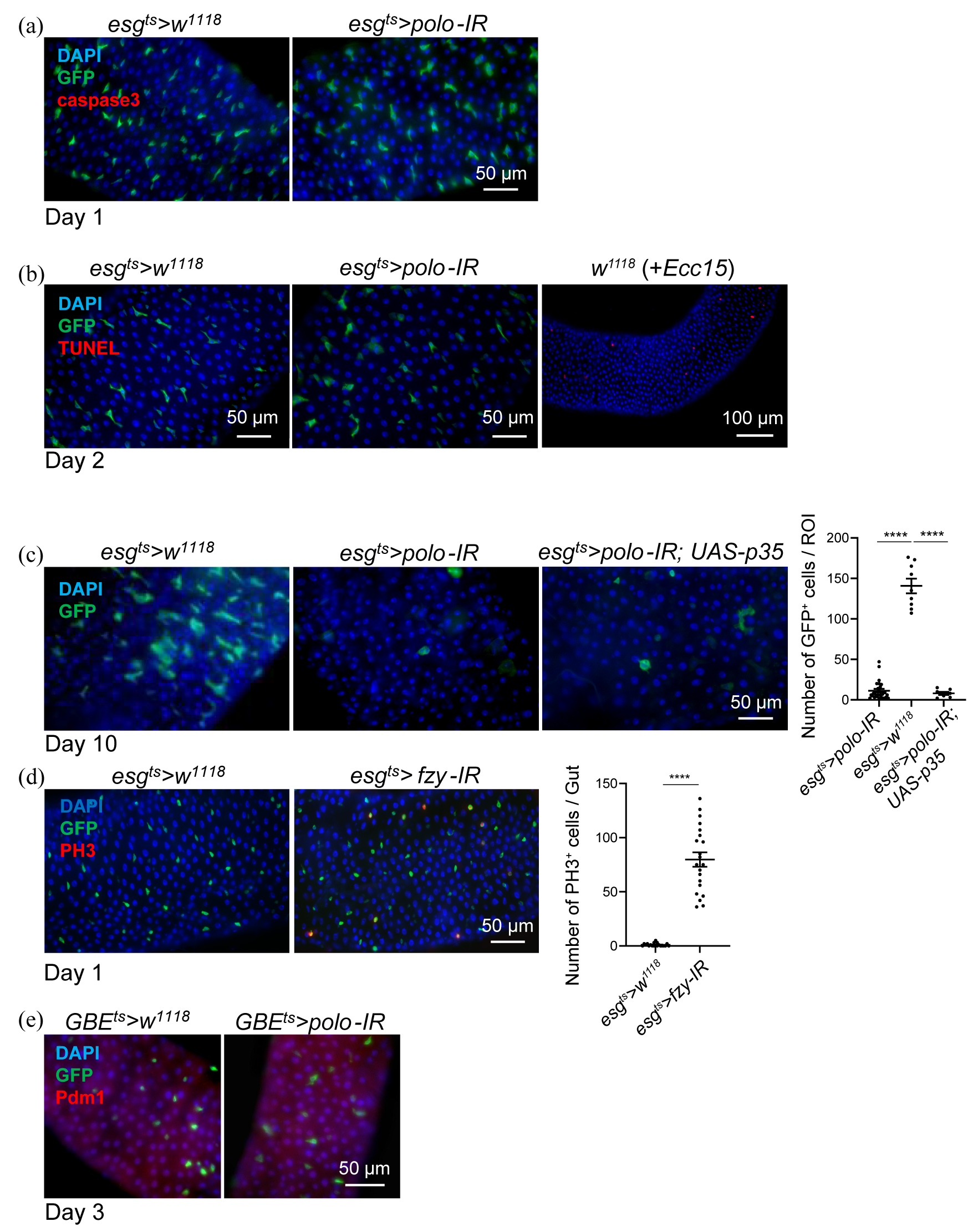

Supplement: jkad084_Supplementary_Data [file jkad084_supplementary_data.zip › Fig._S2_G3-2023-404082.tif]

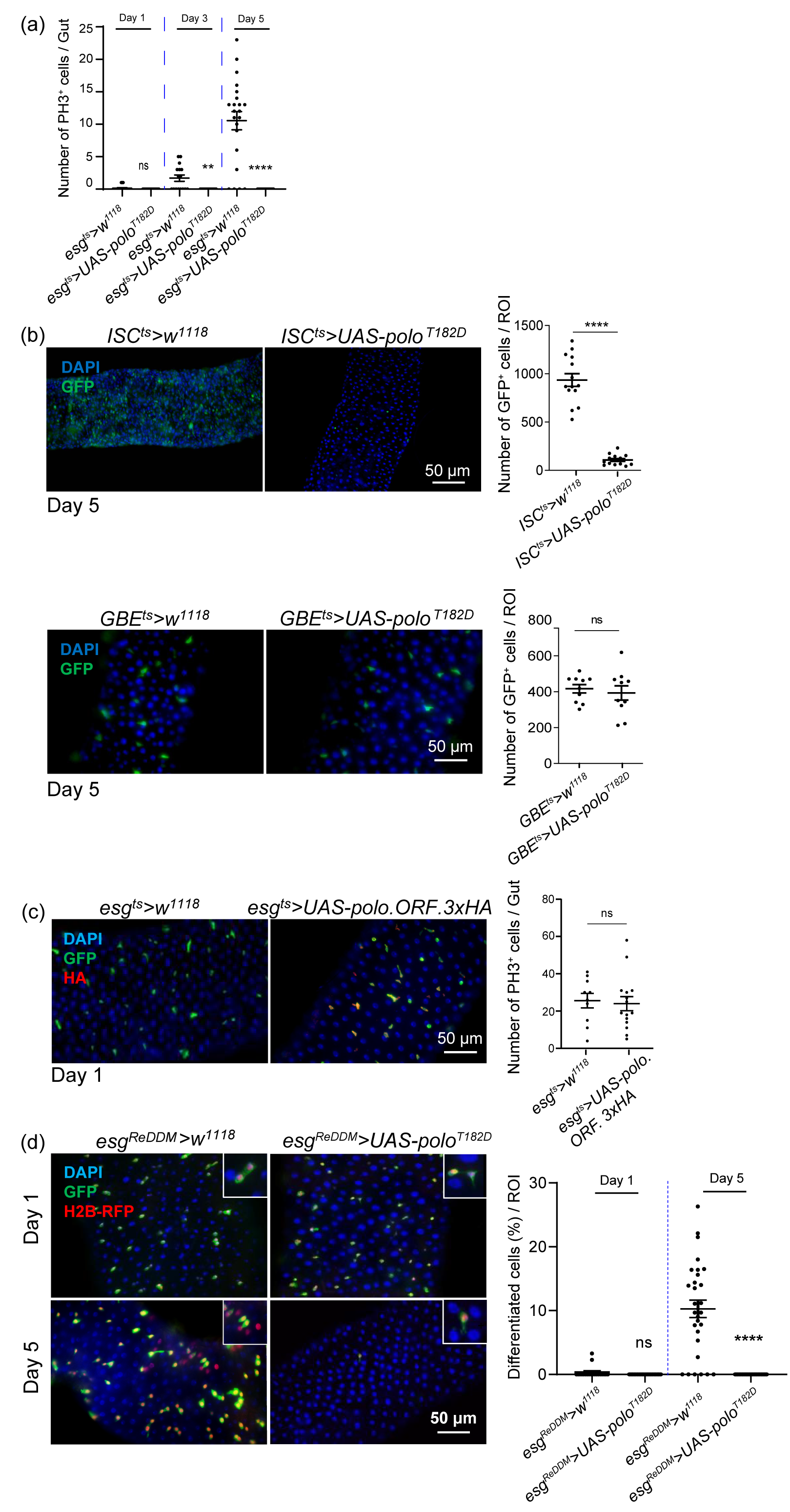

Supplement: jkad084_Supplementary_Data [file jkad084_supplementary_data.zip › Fig._S3_G3-2023-404082.tif]
